# *C. elegans* *rab-18* mutants display reduced lipid content under fed and fasted conditions

**DOI:** 10.17912/micropub.biology.000188

**Published:** 2019-12-27

**Authors:** Zakaria Ratemi, Robert S Kiss, Christian E Rocheleau

**Affiliations:** 1 Department of Biochemistry, McGill University, Montreal, Quebec, Canada; 2 Department of Medicine, Division of Cardiology, McGill University and the Cardiovascular Health Across the Lifespan Program, Research Institute of the McGill University Health Centre, Montreal, Quebec, Canada; 3 Department of Medicine, Division of Endocrinology and Metabolism, McGill University and the Metabolic Disorders and Complications Program, Research Institute of the McGill University Health Centre, Montreal, Quebec, Canada

**Figure 1: C. elegans rab-18 mutants display reduced lipid content under fed and fasted conditions as quantified by Nile Red (NR) and Oil Red O (ORO) staining:**
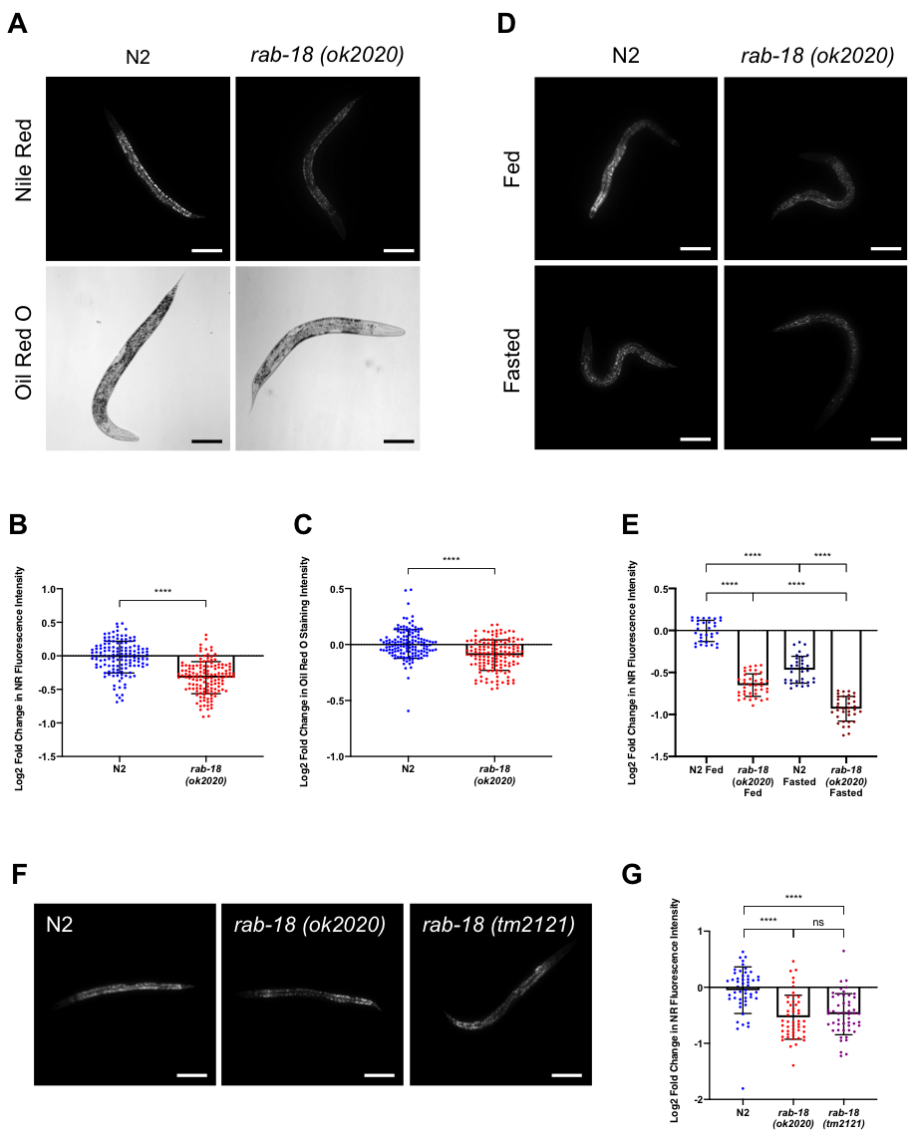
(A) Representative images of NR and ORO stained lipids in fixed wild type (N2) and *rab-18(ok2020)* mutant L4 stage larvae captured at 10X magnification. Scale bar: 100um.  (B) Log2-fold change in NR fluorescence intensity of *rab-18* mutants compared to N2 worms under fed conditions.  (C) Log2-fold change in ORO staining intensity of *rab-18* mutants compared to N2 worms under fed conditions.  (D) Representative images of NR stained lipids in fixed wild type (N2) and *rab-18(ok2020)* mutant worms under fed and fasted (6 hours) conditions at 10X magnification. Scale bar: 100um.  (E) Log2-fold change in NR fluorescence intensities of *rab-18* mutants compared to N2 worms under fed and fasted (6 hours) conditions.  (F) Representative images of NR stained lipids in fixed wild type (N2), *rab-18(ok2020)* , and *rab-18(tm2121)* mutant worms captured at 10X magnification. Scale bar: 100um.  (G) Log2-fold change in NR fluorescence intensities of *rab-18(ok2020)* and *rab-18(tm2121)* mutants compared to N2 worms under fed conditions.  Statistical analysis was performed with Student’s t-test and with a one-way analysis of variance (ANOVA)****p<0.0001. Error bars represent S.D.

## Description

The excessive storage of neutral lipids in lipid droplets (LDs) is a consequence of excess dietary nutrient uptake and the primary cause of major metabolic disorders, including obesity, diabetes, and atherosclerosis (Eckel *et al* ., 2005).  Several studies investigating proteins associated with the monolayer surface of LDs have identified the Rab18 GTPase as a component with a role in the regulation of fat storage and lipid mobilization (Martin *et al* ., 2005; Ozeki *et al* ., 2005). Rab18, a small GTPase protein localized on the surface of LDs, plays a key role in several LD-related processes, including lipogenesis, lipolysis, and lipophagy (Pulido *et al* ., 2011; Schulze *et al* ., 2017).  Although Rab18 has been previously associated with multiple roles in lipid metabolism, including mediating the apposition of LDs to the ER, its fundamental function remains disputed (Ozeki *et al* ., 2005).  To better understand the function of Rab18 in fat control at the organismal level, we proceeded to investigate the effect of *rab-18* knockout on overall lipid abundance in *C. elegans* .

To determine if RAB-18 regulates fat storage in *C. elegans,* we first investigated the fat content of *rab-18(ok2020)* mutants compared to wild type (N2) worms by performing fixed Nile Red (NR) and Oil Red O (ORO) staining experiments under fed conditions. Each NR and ORO experiment was performed with 5 different samples of N2 and *rab-18(ok2020)* worms and 10 worms were analyzed from each sample.  Three separate NR and ORO experiments were performed for a total of 150 images per strain. *rab-18* mutants displayed a significant reduction in NR fluorescence intensity (Figure 1A, B) and ORO staining intensity (Figure 1A, C) compared to the control N2 worms.  This reduction suggests a significant decrease in lipid content in *rab-18(ok2020)* mutants compared to N2 worms.  The less pronounced reduction in the fat content of *rab-18(ok2020)* mutants observed in ORO experiments is attributed to the lower sensitivity of ORO in quantifying changes of lipid abundance compared to NR (Escorcia *et al* ., 2018).

The fixed NR staining experiment of wild type (N2) and *rab-18(ok2020)* mutant worms was also performed under short-term (6 hours) fasting conditions to determine whether the previously observed decrease in fat content of mutant worms under fed conditions was amplified or reduced under short-term fasting conditions. Moreover, fixed NR staining under fasting conditions serves as an internal control to ensure the accuracy of the staining method as the reduction in LD fat content through lipolysis upon short-term fasting is widely acknowledged in the literature (Srinivasan *et al* ., 2015).  Three separate NR staining experiments were performed and 10-15 images were captured per strain for each experiment. Representative images are shown in Figure 1D and quantified in Figure 1E. A significant reduction in NR fluorescence intensity was observed in *rab-18(ok2020)* mutants as compared to wild type (N2) under fasted conditions, suggesting a significant decrease in lipid content.  Moreover, we demonstrate a decrease in fat content under short-term (6 hours) starving conditions in both wild type and *rab-18(ok2020)* mutant worms, which is in line with findings that short-term fasting depletes body fat stores due to the increased expression of ATGL ( *atgl-1* ) lipase mediating lipolysis (Srinivasan *et al* ., 2015).  Thus, RAB-18 promotes lipid storage under both fed and fasted conditions and is not required for lipolysis during short-term fasting.

To rule out the possibility that background mutations might have affected our results, we performed NR staining experiments using an additional *rab-18* deletion allele, *tm2121.* Five separate NR staining experiments were performed on wild type (N2), *rab-18(ok2020)* , and *rab-18(tm2121)* mutant worms and 10 images were captured per strain in each experiment, with representative images shown in Figure 1F and quantified in Figure 1G.  Like the *rab-18(ok2020)* mutant, *rab-18(tm2121)* worms displayed a significant reduction in lipid staining compared to N2 worms.  There was no statistically significant difference between the reduction in fat content observed in *rab-18(ok2020)* and *rab-18(tm2121)* , which reinforces the notion that the observed phenotype is caused by the deletion of *rab-18* .

To confirm that changes in fat storage are due to loss of *rab-18* rather than differences in animal health, we tested for differences in fertility, viability and growth.  Ten animals per strain were permitted to lay eggs for 8 hours and the number of eggs laid was quantitated as a measure of fertility.  Three days later, the number of animals were counted as a measure of viability, and the number of animals that reached maturity (fertile adults) as a measure of growth.  Relative to wild type N2 ( *n=* 305), the number of eggs laid by *rab-18(ok2020)* and *rab-18(tm2121)* was 90% ( *n=* 273) and 79% ( *n=* 240), viability was 99% and 93% and the number of animals reaching maturity was 86% and 63%, respectively.  Thus, *rab-18(ok2020)* grew significantly slower than N2 ( *P<0.0001* ), while *rab-18(tm2121)* was significantly less fertile ( *P<0.01* ), viable ( *P<0.001* ) and grew more slowly ( *P<0.0001* ) than N2.  Notably, *rab-18(ok2020)* and *rab-18(tm2121)* have significant differences in growth ( *P<0.0001* ) and viability ( *P<0.05* ) that could be due to background mutations in these two independently derived strains.  Since *rab-18(ok2020)* and *rab-18(tm2121)* have the same level of reduced fat staining as compared to wild type (N2),we attribute this to the loss of *rab-18* gene function rather than a difference in health.  Our findings demonstrate a role for the RAB-18 GTPase in regulating fat content under both fed and starved conditions, and establish *C. elegans* as a model for analysis of RAB-18 in the regulation of lipid storage.

## Methods

*C. elegans* worms were maintained at 20°C on nematode growth medium (NGM) seeded with HB101 *E. coli* . For fasting experiments, fed L4 stage worms were transferred to unseeded NGM plates for 6 hours. L4 stage worms were fixed in isopropanol and stained with NR (Invitrogen) or ORO (Sigma-Aldrich) as previously described (Escorcia *et al* ., 2018).  Slides carrying the NR stained worms were visualized under the FITC/GFP channel of an Axio Zeiss A1 compound microscope (Zeiss, Oberkochen, Germany) with a 10X objective. Images were captured using an Axio Cam Mrm camera and Axiovision software (Zeiss) with an exposure of 200ms, and maximal UV strength.  Slides with ORO stained worms were visualized by bright-field microscopy with an exposure of 1ms. An ImageJ macro was used to partially automate as well as to increase the efficiency and accuracy of the quantification of the NR/ORO stained images.

Image Quantification :

Computing of the Log2-fold change in NR/ORO staining intensities of *rab-18* mutants compared to N2 worms was achieved using the equation: *log _2_ (staining intensity of rab-18 mutant worm / average staining intensity of N2).*

**Statistics**

Statistical analysis was done using Graphpad Prism software.  Student’s t-test and with an analysis of variance (ANOVA) was used for fat staining, unpaired t-test for fertility and a Fisher’s exact test for viability and growth.

## Reagents

**Table:** 

**Strain Name**	**Genotype**	**Source**
N2	wild type	Caenorhabditis Genetics Center, University of Minnesota
RB1638	*rab-18(ok2020) III*	Caenorhabditis Genetics Center, University of Minnesota
FX2121	*rab-18(tm2121) III*	Shohei Mitani, National BioResource Project, Japan
